# Extensive complex neocortical movement topography devolves to simple output following experimental stroke in mice

**DOI:** 10.3389/fnsys.2023.1162664

**Published:** 2023-06-07

**Authors:** Cassandra C. Wolsh, Rogers Milton Brown, Andrew R. Brown, Gilbert Andrew Pratt, Jeffery Allen Boychuk

**Affiliations:** Department of Cellular and Integrative Physiology, Joe R. and Teresa Lozano Long School of Medicine, University of Texas Health Science Center at San Antonio, San Antonio, TX, United States

**Keywords:** intracortical microstimulation, middle cerebral artery stroke, motor output, neocortex, motor cortex, complex movement

## Abstract

The neocortex encodes complex and simple motor outputs in all mammalian species that have been tested. Given that changes in neocortical reorganization (and corresponding corticospinal output) have been implicated in long term motor recovery after stroke injury, there remains a need to understand this biology in order to expedite and optimize clinical care. Here, changes in the neocortical topography of complex and simple movement outputs were evaluated in mice following experimental middle cerebral artery occlusion (MCAo). Neocortical motor output was defined using long-duration parameters of intracortical microstimulation (LD-ICMS) based on area and spatial coordinates of separate motor output types to build upon our recent report in uninjured mice. LD-ICMS test sites that elicited complex (multi-joint) movement, simple (single skeletal joint) movement, as well as co-elicited FORELIMB + HINDLIMB responses were detected and recorded. Forelimb reaching behavior was assessed using the single pellet reaching (SPR) task. At 6 weeks post-surgery, behavioral deficits persisted and neocortical territories for separate movements exhibited differences in neocortical area, and spatial location, and differed between MCAo-Injured animals (i.e., the MCAo group) and Sham-Injured animals (i.e., the Control group). MCAo-Injury reduced neocortical area of complex movements while increasing area of simple movements. Limited effects of injury were detected for spatial coordinates of neocortical movements. Significant positive correlations were detected between final SPR performance and either area of complex retract or area of co-occurring FORELIMB + HINDLIMB sites.

## Introduction

Volitional and skilled motor behavior is predominantly controlled by the neocortex and its descending corticospinal tract (CST) to spinal motor-related circuitry. Injury, such as stroke, often damages these structures thereby compromising motor function (Tsao et al., 2022). For 100+ years it has been proposed that beneficial reorganization of spared neocortex, and CST, is possible following injury in a manner that supports motor behavior recovery (Glees and Cole, 1949; Nudo et al., 1996; Liepert et al., 2000). Despite this intriguing possibility, the optimal patterns of beneficial change during recovery from CST injury have remained difficult to determine and may depend on the experimental approaches and treatments undertaken to learn about them. Here, complex and simple neocortical motor outputs were studied in a mouse model of middle cerebral artery occlusion (MCAo) to help understand the types of functional neocortical and CST organization and output possible during these recovery processes.

Neocortex of mammalian species possesses a remarkable capacity to produce complex motor output. For example, intracortical microstimulation (ICMS) consisting of relatively longer durations of pulse trains (LD-ICMS, ∼500 ms) has been further advanced in recent years by Graziano et al. (2002, 2005) to demonstrate neocortical complex motor output (Aflalo and Graziano, 2006; Graziano, 2016). In mammals, LD-ICMS readily and consistently evokes complex motor output that spans multiple joints or axial muscles (i.e., complex movements) during stimulation pulse trains that match the temporal time-scale and appearance of wake-behaving motor control. All mammalian species tested with LD-ICMS exhibit complex movements including: macaques (Graziano et al., 2002; Gharbawie et al., 2011a,b; Griffin et al., 2014; Baldwin et al., 2018), capuchins (Mayer et al., 2019), owl and squirrel monkeys (Gharbawie et al., 2011a,b; Stepniewska et al., 2014), galagos (Stepniewska et al., 2005; Cooke et al., 2015), squirrels (Cooke et al., 2012), tree shrews (Baldwin et al., 2017), and rats (Ramanathan et al., 2006; Bonazzi et al., 2013; Brown and Teskey, 2014; Halley et al., 2020).

Studies reporting changes in complex neocortical movement after brain insult, and in the laboratory mouse (Harrison et al., 2012; Brown et al., 2023), are less common. In the rat, complex neocortical motor control following bilateral electrocauterization lesions results in a highly specific form of reorganization that correlates with behavioral performance (Ramanathan et al., 2006). Sensorimotor deprivation, by forelimb immobility cast in rats, reduces complex neocortical motor output in a remarkably reversible manner when immobility cast is subsequently removed (Budri et al., 2014). Reversible cortical cooling in rats, targeted to neocortical territories containing complex movement, produces temporary impairments in forelimb motor function (Brown and Teskey, 2014). As a separate form of complex neocortical motor output, Starkey et al. (2012) observed a positive correlation between behavioral recovery and the neocortical region occupied by FORELIMB + HINDLIMB overlap in rats given ischemic damage by intracortical endothelin-1 injections into neocortex. Together these studies support that beneficial forms reorganization of complex neocortical motor output are possible after acquired brain injury.

Our research group has recently tested laboratory mice to help define several types of complex movement (i.e., ADVANCE, ELEVATE, RETRACT AND DIG), several types of simple movement involving single joints (i.e., DIGIT, WRIST, ELBOW, SHOULDER), additional test sites of forelimb that also co-exhibited hindlimb movement (i.e., FORELIMB + HINDLIMB), in addition to non-forelimb or non-responsive regions (Brown et al., 2023). The present study compared neocortical movement types and their topography in mice given sham-injury or MCAo-Injury wherein ischemic damage was targeted to this blood vessel, rather than a specific region of the neocortical motor map, and resulting forelimb reaching recovery remained comparatively limited across 6 weeks of post-injury assessment. Forelimb reaching behavior was measured using the single pellet reaching (SPR) task (Whishaw and Pellis, 1990; Boychuk et al., 2011; Brown and Teskey, 2014; Boychuk et al., 2017). Area and spatial topography of the aforementioned LD-ICMS-evoked neocortical outputs (complex forelimb, simple forelimb, co-occurring FORELIMB + HINDLIMB, and non-forelimb) were examined. Correlational testing was performed to identify any neocortical motor properties that paralleled behavioral recovery as assessed by the SPR task.

Since stroke injury may be a damaging event to neurons and their synaptic connections this study hypothesized that the area of complex and simple neocortical movement outputs would be equally reduced due to experimental MCAo. Unexpectedly, MCAo reduced the neocortical area of complex motor outputs (including regions of complex forelimb and of overlap of forelimb and hindlimb output) yet increased the neocortical area of simple motor outputs. These changes in motor output area were not paralleled by large shifts in their spatial locations between sham-injury and MCAo-Injury conditions. Area of complex RETRACT and of co-occurring FORELIMB + HINDLIMB sites exhibited significant positive correlations with final SPR performance. The observed changes in neocortical motor outputs may help inform on this biology’s capacity to change in beneficial ways during recovery from brain injury. Combinations of long-standing and emerging *in vivo*, *in vitro* and *in silico* approaches are needed to further advance the understanding of these systems in order to help improve human health and care.

## Materials and methods

### Subjects

Experimentation was performed on 18 male C57BL/6N adult mice (14–16 wk old) purchased from the Jackson Laboratory (Bar Harbor, ME). Mice were housed with same-sex littermates (*n* = 3–5/cage) in clear polycarbonate cages with sawdust bedding and enrichment toys on a 12 h light/dark cycle at the University of Texas Health Science Center at San Antonio (UTHSCSA). Standard mouse chow and water were provided *ad libitum* except that mouse chow was equally removed for all groups tested 2–3 h prior to SPR training. Female mice were not tested here due to the complexity of experimental design and possible effects on experimental stroke injury whereas this important biology merits dedicated assessment. All experimental procedures were approved by the UTHSCSA institutional animal care and use committee (protocol 20180100AR).

### Single pellet reaching (SPR) task

Training, training boxes and analysis of mouse behavior were implemented according to previous studies (Whishaw and Pellis, 1990; Boychuk et al., 2011; Brown and Teskey, 2014; Boychuk et al., 2017). Clear Plexiglass chambers (height: 38.2 cm × width: 14 cm × depth: 33 cm) were fabricated to match previous SPR testing in mice (Regal Plastics, TX). A 1 cm wide vertical hole was made in the front of the chamber from 1.6 cm above the floor to 17.3 cm above the floor. A 3 cm tall shelf (width: 12.3 cm × depth: 5.2 cm) was placed on the outside of the chamber to allow mice to reach the shelf through the hole on the front of the chamber. Mice were familiarized to dustless precision pellets (F0071; Bioserv, NJ) by introducing the pellets to the cages several days before beginning training. Mice were then placed in the chambers for 30 min with pellets on the floor to familiarize the mice to the environment. Following 3 sessions of familiarization, pellets were placed on the shelf to encourage reaching by either limb through the hole in the chamber for the pellet. Preferred handedness was determined for each mouse as the first forelimb to produce 10 successful reaches. Successful reaches required the mouse to grasp the pellet with its digits, bring it into the chamber, and consume it without dropping it at any point during the reaching movement. Mice were trained to attempt a single reach attempt at every approach to the front of the chamber and all reach attempts (forelimb excursions that extended beyond the reaching window) were recorded. The pellet was then moved to the side of the platform opposite of the dominant forelimb to encourage reaching with that forelimb. The number of reaches attempts and successful reaches were video recorded and quantified for all daily 30-min sessions (1–3 pm; Mondays, Wednesdays, and Fridays).

### Middle cerebral artery occlusion (MCAo) injury

Mice were divided into sham (*n* = 9) and MCAo injury groups (*n* = 9) by equally balancing SPR performance (determined as the performance average during the final 3 days of assessment). Animals were anesthetized with isoflurane during stroke and sham surgery. Both groups received retro-orbital injections of Rose Bengal Dye (50 uL; 20 mg/ml, Sigma). The distal MCA is exposed by moving the temporal muscle and providing a burr hole below the zygomatic arch. Continuous light stimulation of the dye is delivered to the burr hole using a custom fabricated optical cannula (RJPFF4; Thor Labs, NJ) attached to a green laser (565 nm; OEM Laser Systems) controlled by a pulse generator (Model 2100; A-M Systems, WA) wherein optical power at electrode tip was adjusted to 3 mW for each surgery (RS121C and PM100D; Thor Labs, NJ). Stimulation of the dye activates clotting factors resulting in a visible permanent clot at the distal MCA. All subjects received post-operative monitoring and care (Buprenorphine HCL; 1 mg/kg). Sham surgery mice were given all aspects except no optical light exposure. This injury may damage the coupling of crucial sensory and motor information processing.

### Electrophysiological mapping

Standard LD-ICMS techniques developed for the mouse were used to generate motor maps of neocortical caudal (CFA) and rostral (RFA) forelimb areas (Neafsey et al., 1986). Whereas a single time point was selected for LD-ICMS analysis here additional timepoints would contribute to understanding the temporally complex set of biological changes of stroke recovery. Mice (Sham, 27.27 ± 2.33 g; MCAo, 25.97 ± 3.52 g) were anesthetized with ketamine hydrochloride (150 mg/kg, i.p.) and xylazine (10 mg/kg, i.p.) and the preferred forelimb identified on the SPR task was shaved. Mice were then secured in a stereotaxic frame on an aluminum block (7.5 L × 2.5 W × 2.0 H cm) to elevate the torso and allow free range of forelimb movement. A feedback-controlled heating pad-maintained core body temperature at ∼37.5°C. Supplemental injections of either ketamine (25 mg/kg) or a mixture of ketamine (10 mg/kg) and xylazine (1 mg/kg) were given as required throughout surgery to maintain a constant level of anesthesia as determined by monitoring vibrissae whisking, breathing rate, and cutaneous reflexes in response to a gentle foot/tail pinch (Table 1). A craniotomy was performed over the sensorimotor cortex opposite each animal’s preferred reaching forelimb and physiological saline heated to body temperature was used to cover the cranial window prevent cortical desiccation. An image of the exposed portion of the brain was captured using a digital camera coupled to a stereomicroscope (Olympus SZ61; Waltham, MA) and displayed on a personal computer. A grid of 500 μm squares was then overlaid on the digital image using imaging software (CorelDRAW; Ottawa, ON) and was calibrated to bregma using sagittal and frontal suture intersection coordinates obtained prior to the craniotomy. Electrode penetrations were performed at the intersections of the grid lines and in the center of each square to give an interpenetration distance of 354 μm, except when located over a blood vessel in which case a penetration was not performed or was performed at the minimal safe distance relative to the vessel.

**TABLE 1:** Analysis of spared tissue and long-duration parameters of intracortical microstimulation (LD-ICMS) surgical anesthetic between groups.

Condition	Mean	SD	SEM	Corrected *p*-value
**Spared tissue: percent area of surgery hemisphere to non-surgery hemisphere**				
Sham (*n* = 9)	100.35	8.21	2.74	0.032*
MCAo (*n* = 9)	89.61	13.64	4.55	
**Ketamine and xylazine administration during LD-ICMS surgery (mg/kg/hr)**				
Ketamine-sham (*n* = 7)	1.089	0.46	0.17	0.28
Ketamine-MCAo (*n* = 7)	1.20	0.20	0.076	
Xylazine-sham (*n* = 7)	0.084	0.014	0.0055	0.37
Xylazine-MCAo (*n* = 7)	0.080	0.026	0.0099	

Asterisk indicates significance: *p ≤ 0.05.

Glass-coated platinum/iridium microelectrodes with an input impedance of 0.5 ± 0.1 MΩ (1000 Hz, 10 nA) were used (FHC Inc., Bowdoin, ME). Electrodes were guided into the neocortex to a depth of 800 μm via microdrive (Narishige, Tokyo), corresponding to the deep layer V (Tennant et al., 2011), and adjusted to maximize the amplitude of evoked responses. Movements can be readily elicited within a large depth range from surface (650–1000 μm) in the mouse with negligible effect on their nature or threshold (Young et al., 2011). An isolated pulse stimulator (Model 2100, A-M Systems; Carlsborg, WA) was used to deliver electrical current consisting of 500 ms trains of cathodal-leading 200 μs biphasic pulses delivered at a frequency of 333 Hz and an intensity of 100 μA consistent with previous studies (Graziano et al., 2002; Ramanathan et al., 2006; Bonazzi et al., 2013; Brown and Teskey, 2014; Budri et al., 2014; Brown et al., 2020; Singleton et al., 2021; Brown et al., 2023). Pulse trains were delivered in 0.2 Hz frequencies up to a maximum of 6 per site to ensure the stability of evoked responses with stimulation sites evoking movement in ≥50% of trials considered responsive.

Long-duration parameters of intracortical microstimulation testing and quantification was performed as described recently by the laboratory (Brown et al., 2023). Mice were supported in a limb-free prone position with the wrist palm-down and digit, wrist, elbow, shoulder joints semi-flexed. Between stimulation trials, forelimb resting position was reset. Following the first stimulation site aimed at the center of the forelimb motor cortex, subsequent stimulations followed in the parasagittal direction until either a non-forelimb or non-responsive point was observed. Mapping continued in a row-by-row fashion on the overlaid grid until the border of the forelimb representation consisting of either a non-forelimb or non-responsive points was encountered. Forelimb map boundaries were defined by mapping all sites adjacent to a forelimb-responsive site as either non-responsive or non-forelimb. Throughout the surgery, anesthetic levels were monitored by verifying evoked movements in previously defined positive-response sites and surrounding non-responsive and non-forelimb points that define the outside border of positive-response sites. Only complete LD-ICMS assessments wherein all forelimb-responsive sites were identified by these criteria were included in subsequent analyses.

Movements were monitored visually during electrophysiological mapping and video-recorded from a left side view for offline assessment (100 frames/s, 2 ms shutter, Matrix Vision; Oppenwiler, Germany) with anatomical reference markers placed to identify the proximal termination of the humerus (shoulder), elbow, wrist, metacarpophalangeal joints, and the tip of the fourth digit with titanium dioxide paste to assist movement detection (Simi Motion; Unterschleißheim, Germany). A light-emitting diode synchronized with stimulator output was fixed to the stereotaxic frame in the camera field of view to determine stimulation onset. Evoked forelimb movements contralateral to the stimulated hemisphere were characterized as either complex when involving multiple joints during the stimulation or simple when involving a single joint (flexion/extension of the digit, wrist, elbow, or shoulder) according to previously defined criteria (Brown and Teskey, 2014; Brown et al., 2023). Forelimb movements that co-occurred with non-forelimb movements were classified as forelimb responses for areal measurements and the co-occurrence of hindlimb responses was recorded and quantified to determine FORELIMB + HINDLIMB overlap. The areal map size for each movement was calculated with every stimulation site corresponding to 0.125 mm^2^ of the cortical surface (354 μm X 354 μm).

### Stroke injury histology and measurement

Following extraction, brains were immediately place in 4% paraformaldehyde solution in 0.1 M sodium phosphate buffer. After a fixation period of at least 1 week, full brains were sectioned at 30-μm (Leica CM1860). Sections were stored individually in 24 well plates of 0.1M sodium phosphate buffer. Samples of sections were taken in a 1 in 6 series and stained with cresyl violet to help visualize regions of ischemic damage. Sections were digitally scanned on slides and 6 sections (spaced 180 um apart) were selected for analyses in the anteroposterior axis from each subject (approximate bregma levels of 0.54, 0.36, 0.18, 0, −0.18, −0.36 mm). The area of injury was calculated with Image J software by separately quantifying the area of spared tissue in the hemispheres ipsilateral and contralateral to MCAo or sham-injury (Abràmoff et al., 2004). Measured areas were then averaged and analyzed as the percentage affected to unaffected side [(Mean area ipsilateral hemisphere/Mean area contralateral hemisphere) × 100] to account for inter-individual differences in brain size.

### Statistical analyses

Here, comparisons were made between sham-injury (i.e., Control) and permanent ischemic injury using middle cerebral artery occlusion (i.e., MCAo). Mean difference in cortical movement representation area, stimulation site location, and anesthetic administered as a function of body weight and mapping duration between groups were assessed with *t*-tests using the Holm-Sidak procedure for multiple-comparison testing or unpaired *t*-tests as appropriate for number of factors. Behavior results were assessed with repeated measures 2-Way ANOVA followed by Fisher’s LSD multiple comparison to detect differences between group. Correlations were tested between individual LD-ICMS response types and average percent reach accuracy from final 4 sessions before mapping. Comparison of the variance in mean movement representation X and Y coordinates between simple and complex movements were performed with a *t*-test. Mann-Whitney test was used as a non- parametric alternative for instances of unequal variance. Statistical analyses were performed with GraphPad Prism 9 (GraphPad Software, La Jolla, CA). An experiment-wide α level of 0.05 was used and asterisks in figures and tables indicate significance: *p ≤ 0.05, ^**^p ≤ 0.01, ^***^p ≤ 0.001. Reported *p*-values are adjusted for multiple comparisons. Data are reported as mean ± SD with raw data points.

## Results

### Spared tissue and forelimb motor behavior

**FIGURE 1:**
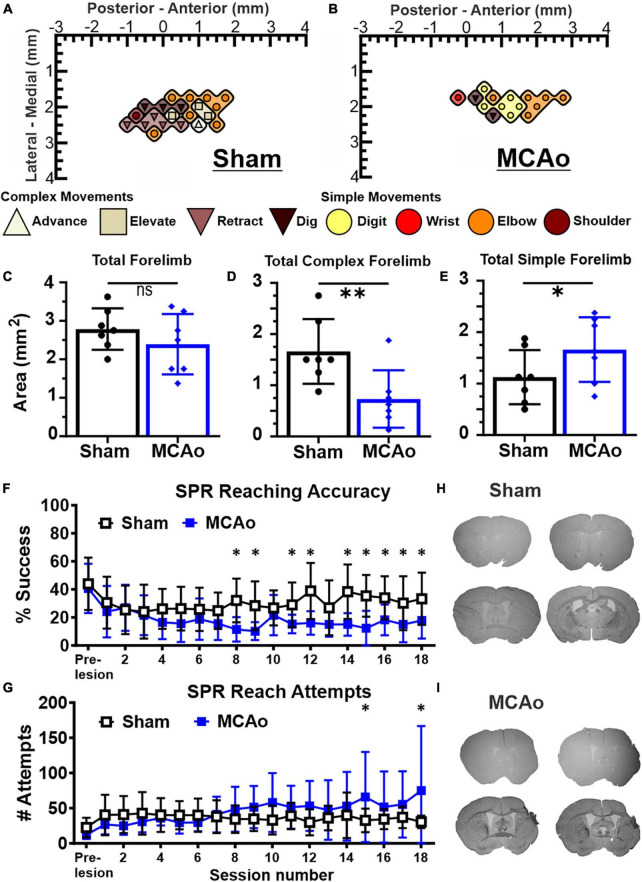
Middle cerebral artery occlusion (MCAo) mice exhibit forelimb reaching deficits and a decrease in neocortical area for complex motor output that is paralleled by an increase in neocortical area for simple motor output. Long-duration intracortical microstimulation (LD-ICMS) produces simple and complex movements of the mouse forelimb. Representative images of the neocortical representation of complex and simple movement in a mouse given sham injury **(A)** and a mouse given injury by MCAo **(B)**. Bregma is represented as a 0 value in the anterior-posterior axis. The neocortical area for total forelimb movement does not differ between sham-injury (*n* = 7) and MCAo-injury (*n* = 7) conditions **(C)**. The neocortical area for complex forelimb movement is significantly reduced in MCAo-injured mice compared to sham-injury **(D)** whereas MCAo-injured animals exhibit a larger neocortical area for simple forelimb movement **(E)**. Single pellet reach (SPR) training was performed on both surgery groups for 18 sessions, with reaching accuracy **(F)** and number of reach attempts **(G)** reported for each group (Sham *n* = 9, MCAo *n* = 9). MCAo-injury persistently reduced forelimb reaching accuracy and resulted in inconsistent increases in the number of reach attempts. Representative sections from sham **(H)** and MCAo **(I)** conditions demonstrate significant neocortical damage due to MCAo-Injury (see Table 1). Mean values ± SD and individual points are provided in panels **(C–E)**. *P ≤ 0.05, ^**^P ≤ 0.01.

Histological injury, resulting from sham-injury or MCAo-Injury, was quantified as area of spared tissue that was subsequently normalized as percent of hemisphere given surgery relative to the contralateral hemisphere to account for inter-individual differences in brain size. The MCAo-Injured group (mean: 89.6 ± 13.6%) exhibited significantly less spared tissue than the Sham-Injured group (mean: 100.3 ± 8.2%) [*T*(16) = 2.03; *p* = 0.032] (Figures 1H, I and Table 1).

For *in vivo* behavior, the single pellet reaching (SPR) task was used to measure the number of reach attempts and the percent of successful reaches as a quantification of upper extremity performance, following sham-injury or MCAo-Injury (Figures 1F, G). MCAo-Injury did not have a significant overall effect on the number of reach attempts (2-Way RM ANOVA; *F*(1,16) = 0.42, *p* = 0.53) whereas an overall effect of training session (2-Way RM ANOVA; *F*(17,272) = 2.32, *p* = 0.0026) and a significant interaction between session number and experimental condition (2-Way RM ANOVA; *F*(17,272) = 2.58, *p* = 0.0007) were detected for reach attempts. Baseline (pre-injury) number of reach attempts did not significantly differ between Sham-Injured (mean: 22.52 ± 4.62) and MCAo-Injured animals (mean: 12.22 ± 1.99; Fisher’s LSD, *p* = 0.44). Whereas most behavioral sessions did not differ between groups, MCAo-Injured animals made significantly more reach attempts (mean: 65.89 ± 21.34) than Sham-Injured controls during session 15 (mean: 32.67 ± 5.59; Fisher’s LSD, *p* = 0.014) thereby suggesting that they were participating in the task and were capable of producing at least some of the movements necessary for its completion; albeit with less accuracy (Figures 1F, G).

For percent successful reaches, an overall effect of injury condition (2-Way RM ANOVA; *F*(1,16) = 5.01, *p* = 0.040), reaching session (2-Way RM ANOVA; *F*(17,272) = 4.88, *p* ≤ 0.0001), and their interaction were significant (2-Way RM ANOVA; *F*(17,272) = 2.62, *p* = 0.0006). Baseline (pre-injury) percent success did not significantly differ between the MCAo-Injured (mean: 40.73 ± 5.86%) and Sham-Injured groups (mean: 43.98 ± 6.23%; Fisher’s LSD, *p* = 0.64). The MCAo-Injury group exhibited reduced reaching accuracy during post-injury sessions 8, 9, 11, 12, 14–17 (Figure 1F). In summary, MCAo-Injured mice were participating in the SPR task but were less accurate during multiple training sessions and failed to achieve the same performance as pre-injury or sham-injury. It is possible that MCAo-Injured animals here possess specific deficits in satiety, motivation, and sensory function in manner that is detrimental to their SPR accuracy. Following 6 weeks of behavioral assessment we then determined the neocortical representation of complex and simple movement on these 2 experimental groups.

### Neocortical movement types

**FIGURE 2:**
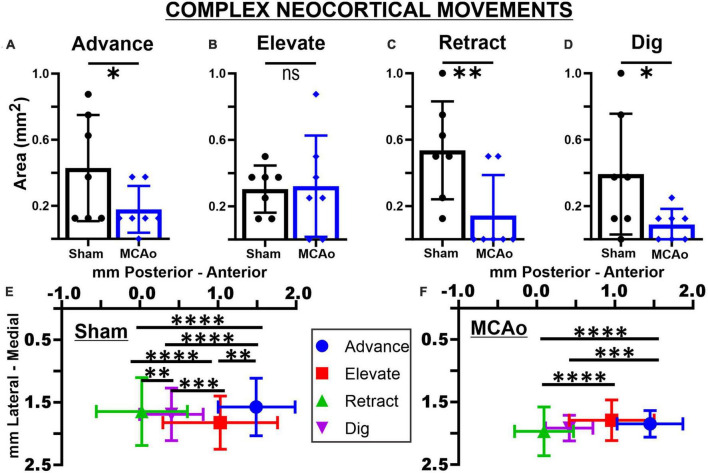
Middle cerebral artery occlusion differentially affects neocortical area and spatial topography of individual complex forelimb movement types. Neocortical areas of complex forelimb ADVANCE **(A)**, RETRACT **(C)**, and DIG **(D)** are reduced after MCAo-injury (*n* = 7) relative to sham-injury (*n* = 7) whereas area of ELEVATE **(B)** is not significantly affected. Mean values ± SD and individual points are provided in panels **(A–D)**. Each complex movement type, at each test site, was assessed by its X and Y coordinates (mm) to derive their mean topographical locations for sham-injured **(E)** and MCAo-injured **(F)** animals (bars represent SD; see the section “Materials and methods”). No significant between-group differences were detected for X or Y coordinates of individual movement types (Tables 2, 3). For X coordinates of the sham-injury group, each type of complex movement exhibited a significantly different mean neocortical location from each other (Tables 2, 4). For X coordinates of the MCAo-injured group, complex movement types exhibited a significantly different mean neocortical location from each other except no significant differences were detected between comparisons of RETRACT-DIG, ADVANCE-ELEVATE and ELEVATE-DIG. No significant within-group differences were detected for Y coordinates. *P ≤ 0.05, ^**^P ≤ 0.01, ^***^P ≤ 0.001, ^****^P ≤ 0.0001. Bregma is represented as a 0 value in the anterior-posterior axis.

**FIGURE 3:**
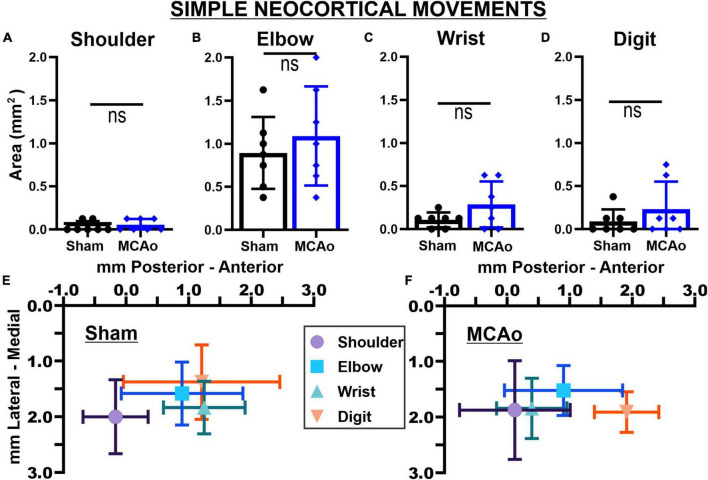
Middle cerebral artery occlusion-Injury fails to significantly affect neocortical area and spatial topography of individual simple forelimb movement types. Simple movements observed during LD-ICMS did not statistical differ between sham-injury (*n* = 7) and MCAo-Injury (*n* = 7) groups: SHOULDER **(A)**, ELBOW **(B)**, WRIST **(C)**, and DIGIT **(D)**. Mean values ± SD and individual points are provided in panels **(A–D)**. Each complex movement type, at each test site, was assessed by its X and Y coordinates (mm) to derive their mean topographical locations for sham-injured **(E)** and MCAo-injured **(F)** animals (bars represent SD; see the section “Materials and methods”). No significant differences were detected for X or Y coordinates between-groups (Tables 2, 3) or within-groups (Tables 2, 4). Bregma is represented as a 0 value in the anterior-posterior axis.

Long-duration parameters of intracortical microstimulation was performed in mice in a manner consistent with our recent report (Brown et al., 2023) to establish complex and simple movement types and neocortical topography within sham-injury control and MCA-injured conditions. In all cases, the hemisphere contralateral to the preferred reaching forelimb of each animal was analyzed. A total of 843 electrode penetrations, targeted to sensorimotor cortex located ipsilateral to injury, were performed in 14 mice (sham-injury *n* = 7; MCAo-Injury *n* = 7). Responses comprised 301 forelimb, 62 non-forelimb, and 480 non-responsive stimulation sites to characterize evoked forelimb movements and to delineate their cortical topography with LD-ICMS. All movement types were analyzed both for neocortical area and spatial topography based on XY cartesian coordinates (location data presented in Figures 2, 3). A comparison of the amount administered ketamine [*T*(12) = 0.58; *p* = 0.28] and xylazine [*T*(12) = 0.35; *p* = 0.37] failed to detect significant differences between experimental groups (Table 1).

Both complex and simple forelimb movements were evoked from stimulation of the sensorimotor cortex in a manner consistent with our recent report (Figures 1A, B; Brown et al., 2023). In brief, we observed 3 complex forelimb movements comprising movement across multiple joints that are similar in Long Evans rats (Brown and Teskey, 2014) and mice (Brown et al., 2023): ELEVATE - characterized as a dorsal displacement of the forelimb involving flexion of the elbow and extension of the wrist; ADVANCE - characterized as an anterior displacement of the forelimb involving flexion of the shoulder and extension of the wrist; and RETRACT - characterized as a posterior displacement of the forelimb involving flexion of the elbow and extension of the shoulder and wrist. We also reobserved a complex movement in the mouse that we previously termed DIG (Brown et al., 2023) that consists of a posteroventral displacement of the forelimb involving extension of the shoulder and elbow with flexion of the wrist. Simple neocortical forelimb movements generated by LD-ICMS consisted of single-joint flexions, or extensions, of SHOULDER, ELBOW, WRIST, AND DIGIT that temporally extended throughout the stimuli trains but otherwise closely matched the outputs observed during established ∼40 msec train ICMS (Asanuma et al., 1968; Kleim et al., 1998; Brown and Teskey, 2014; Brown et al., 2020; Brown et al., 2023). Certain neocortical regions exhibited combinations of forelimb (complex or simple) and hindlimb motor responses, i.e., overlap of FORELIMB + HINDLIMB movements; these overlapping outputs were recorded and analyzed.

### Area of total, complex, and simple neocortical motor outputs

The area (mm^2^) of the mouse neocortex occupied by LD-ICMS-evoked forelimb outputs was quantified for total forelimb, total complex forelimb, total simple forelimb in addition to their respective movement sub-types (Figures 1A–E). The total area of all forelimb movements between the MCAo-Injured mice (mean: 2.39 ± 0.30) was not significantly different from the total forelimb area in the Sham-Injured controls (mean: 2.79 ± 0.20), [*T*(12) = 1.09; *p* = 0.15]. Total neocortical area for complex forelimb movements was significantly reduced in the MCAo-Injured group (*n* = 7; mean = 0.73 ± 0.21) in comparison to Sham-Injured controls (*n* = 7; mean = 1.66 ± 0.24); [*T*(12) = 2.91; *p* = 0.0066]. In contrast, total motor map area for simple forelimb movements was significantly increased in the MCAo group (mean: 1.66 ± 0.24) in comparison to Sham-Injured controls (mean: 1.13 ± 0.20), [*T*(12) = 1.73; *p* = 0.05]. Together, total neocortical forelimb area was statistically unaltered by MCAo whereas a reduction in area of complex movement and parallel increase in area of simple movement were observed. Importantly, neither Sham-Injured nor MCAo-Injured neocortical topography exhibited sites of complex forelimb movement that were directly surrounded by non-responsive sites, or directly surrounded by simple forelimb movements, thus suggesting that discrete “islands” of complex forelimb movement were not formed by these experimental conditions.

### Area of separate complex neocortical motor outputs

The areas of distinct individual complex movements exhibited differential responses to MCAo-Injury. Based on comparing MCAo-Injured animals (mean: 0.32 ± 0.12) to Sham-Injured controls (mean: 0.30 ± 0.054), ELEVATE was the only complex movement in which the area was not significantly reduced with injury [*T*(12) = 0.14; *p* = 0.45] (Figures 2A–D). The area of ADVANCE [*T*(12) = 1.88; *p* = 0.042], RETRACT [*T*(12) = 2.72; *p* = 0.0094], and DIG [*T*(12) = 1.73; *p* = 0.027, Mann-Whitney *p* = 0.038] were significantly reduced in the MCAo-Injury group (ADVANCE, mean: 0.18 ± 0.054; RETRACT, mean: 0.14 ± 0.092; DIG, mean: 0.089 ± 0.036) compared to Sham-Injured controls (ADVANCE, mean: 0.43 ± 0.12; RETRACT, mean: 0.54 ± 0.11; DIG, mean: 0.39 ± 0.14). Thus, most complex movement types exhibited a reduction in neocortical area following MCAo whereas a similar response was not detected for ELEVATE.

### Spatial coordinates: all complex and simple neocortical motor outputs

Each neocortical movement type, at each test site, was subsequently assessed by its X and Y coordinates (mm) to derive their topographical locations (mean ± SD) for Sham-Injured (Figures 2E, 3E) and MCAo-Injured (Figures 2F, 3F) animals (Tables 2–4). X and Y coordinates were statistically compared separately and were analyzed in several ways (overall main effects presented in Table 2). Between-group-*post hoc* comparisons were used to test for an effect of injury on spatial location (Table 3) for either X or Y neocortical locations of LD-ICMS movements. Within-group-*post hoc* comparisons were used to test whether individual movement types differed in either X or Y neocortical locations in either Sham-Injured or MCAo-Injured groups (Table 4). Separate movement types were tested individually and as collapsed categories of either total, complex or simple motor outputs.

**TABLE 2:** Spatial coordinates of collapsed total, complex and simple neocortical motor output.

	X-coordinates (total)	Y-coordinates (total)	X-coordinates (complex)	Y-coordinates (complex)	X-coordinates (simple)	Y-coordinates (simple)
**2 WAY ANOVA main effect *P*-values**						
Interaction	0.079	0.25	0.96	0.36	0.19	0.16
Injury condition	0.79	0.056	0.94	0.021*	0.28	0.54
Movement type	0.76	0.014**	<0.0001****	0.79	0.17	0.071

Asterisks indicate significance: *p ≤ 0.05, **p ≤ 0.01, ****p ≤ 0.0001.

**TABLE 3:** Between-subject spatial coordinates of total, individual complex and individual simple neocortical motor output (separated by X and Y coordinates).

	Sham mean ± SD (mm)	MCAo mean ± SD (mm)	Corrected *p*-value
**Between group X-coordinates: neocortical spatial location**			
Total	0.78 ± 0.88	0.75 ± 0.82	0.37
Complex	0.70 ± 0.80	0.85 ± 0.67	0.31
Advance	1.49 ± 0.49	1.45 ± 0.42	0.99
Elevate	1.03 ± 0.73	0.96 ± 0.54	0.99
Retract	0.026 ± 0.58	0.094 ± 0.38	0.99
Dig	0.41 ± 0.41	0.42 ± 0.30	0.99
Simple	0.91 ± 0.98	0.70 ± 0.88	0.25
Shoulder	−0.17 ± 0.52	0.13 ± 0.88	0.92
Elbow	0.90 ± 0.97	0.90 ± 0.94	0.97
Wrist	1.25 ± 0.65	0.40 ± 0.56	0.15
Digit	1.21 ± 1.25	0.66 ± 0.52	0.50
**Between group Y-coordinates: neocortical spatial location**			
Total	1.65 ± 0.51	1.72 ± 0.44	0.12
Complex	1.68 ± 0.47	1.86 ± 0.29	0.079
Advance	1.57 ± 0.46	1.85 ± 0.21	0.24
Elevate	1.82 ± 0.42	1.79 ± 0.32	0.83
Retract	1.65 ± 0.54	1.97 ± 0.39	0.22
Dig	1.69 ± 0.42	1.92 ± 0.20	0.44
Simple	1.61 ± 0.57	1.65 ± 0.48	0.57
Shoulder	2.0 ± 0.66	1.88 ± 0.88	0.95
Elbow	1.58 ± 0.56	1.52 ± 0.44	0.89
Wrist	1.83 ± 0.47	1.84 ± 0.54	0.97
Digit	1.38 ± 0.67	1.91 ± 0.36	0.12

Total analyzed with one-tailed *t*-test, individual movements analyzed with 2-way ANOVA between groups and Holms-Sidak correction.

**TABLE 4:** Within-group spatial coordinates of simple versus complex, individual complex and individual simple neocortical motor output (separated by X and Y coordinates).

	X-coordinate within group	Y-coordinate within group
**2-WAY ANOVA complex movements comparison within group**		
**Sham**		
Advance vs. elevate	0.014**	0.34
Advance vs. retract	<0.0001****	0.81
Advance vs. dig	<0.0001****	0.81
Elevate vs. retract	<0.0001****	0.62
Elevate vs. dig	0.001***	0.81
Retract vs. dig	0.014**	0.81
**MCAo**		
Advance vs. elevate	0.059	0.98
Advance vs. retract	<0.0001****	0.98
Advance vs. dig	0.001***	0.98
Elevate vs. retract	0.0009***	0.91
Elevate vs. dig	0.063	0.98
Retract vs. dig	0.26	0.98
**2-way ANOVA simple movements comparison within group**		
**Sham**		
Shoulder vs. elbow	0.17	0.52
Shoulder vs. wrist	0.14	0.64
Shoulder vs. digit	0.14	0.40
Elbow vs. wrist	0.73	0.59
Elbow vs. digit	0.73	0.59
Wrist vs. digit	0.94	0.47
**MCAo**		
Shoulder vs. elbow	0.72	0.80
Shoulder vs. wrist	0.83	0.99
Shoulder vs. digit	0.83	0.99
Elbow vs. wrist	0.17	0.083
Elbow vs. digit	0.83	0.061
Wrist vs. digit	0.83	0.97

Asterisks indicate significance: **p ≤ 0.01, ***p ≤ 0.001, ****p ≤ 0.0001.

In contrast to statistical reporting of neocortical area, spatial findings of neocortical motor output here revealed fewer significant differences (Figures 2E, F). For X coordinates, a significant within-group effect of complex movement type [*F*(3,125) = 36.39, *p* ≤ 0.0001] was detected. For Y coordinates, a significant within-group effect of total movement type (simple + complex) [*F*(1,29) = 6.16, *p* = 0.014] and a significant between-group effect of MCAo-Injury (for complex movement) *F*(1,125) = 5.49, *p* = 0.021] were detected (Table 2). Importantly, all protected and unprotected between-group *post hoc* comparisons, of X or Y coordinates, failed to detect any statistically significant effects (of MCAo-Injury) regarding spatial localization (Table 3). Thus, experimental stroke failed to result in robust effects in the spatial location of either simple or complex neocortical motor outputs.

Within-group *post hoc* comparisons were also performed for spatial coordinates since overall main effects of movement type were detected. Within either sham-injury or MCAo-Injury conditions, individual types of complex movement (Figures 2E, F) were compared to one another or individual types of simple movement (Figures 3E, F) were compared to one another (Table 4). Interestingly, no significant differences were detected for spatial coordinates (X or Y) for any simple movement comparisons. The lack of differences for simple movement types may in part be due to the relatively larger standard deviations of X or Y coordinates. Equally interestingly, no significant differences were detected for spatial Y coordinates for any complex movement comparisons. Instead, *post hoc* comparisons for spatial X coordinates for complex movement comparisons revealed several significant within-group relationships (Table 4). Thus, within conditions tested, the spatial localization of separate motor output types predominantly exhibited significant differences for within-group X coordinates of complex movement.

### Area of separate simple neocortical motor outputs

In contrast to complex motor outputs, the area of distinct individual simple movements were numerically, but not statistically, increased by MCAo-injury: SHOULDER [*T*(12) = 0.52; *p* = 0.31], ELBOW [*T*(12) = 0.73; *p* = 0.24], WRIST [*T*(12) = 1.68; *p* = 0.059], and DIGIT [*T*(12) = 1.09; *p* = 0.15] (Figures 3A–D). Mean values for these simple movements for the MCAo-Injured group were: SHOULDER, mean = 0.054 ± 0.025; ELBOW, mean: 1.09 ± 0.22; WRIST, mean: 0.29 ± 0.101; DIGIT, mean: 0.23 ± 0.12). Mean values for these simple movements for the Sham-Injured controls were: SHOULDER, mean: 0.036 ± 0.023; ELBOW, mean: 0.89 ± 0.16; WRIST, mean: 0.29 ± 0.10; DIGIT, mean: 0.089 ± 0.053. In summary, between-group comparisons (sham-injury versus MCAo-Injury) predominantly identified significant changes in neocortical area whereas within-group comparisons (movement type versus movement type) predominantly identified significant differences in neocortical spatial location.

### Area of overlapping FORELIMB + HINDLIMB neocortical motor outputs and all correlational testing

**FIGURE 4:**
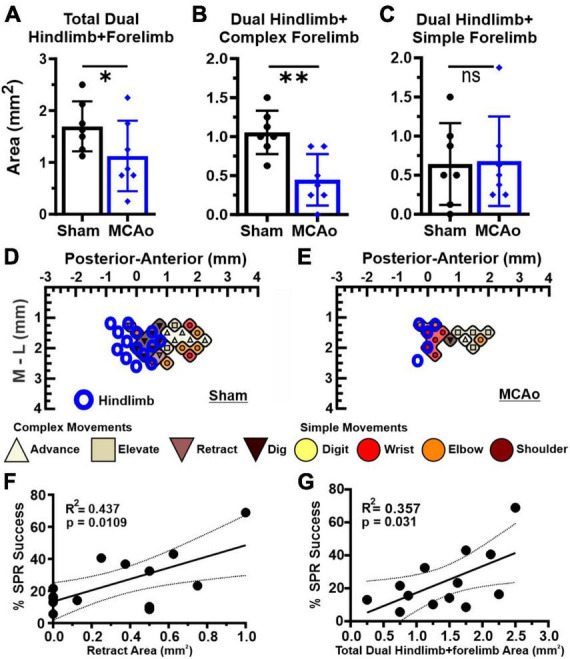
Dual HINDLIMB-FORELIMB movements of neocortex are differentially affected by MCAo-injury whereas 2 aspects of complex neocortically derived motor output exhibited a positive correlation with final SPR behavior. Comparisons were made between groups (sham-injury, *n* = 7; MCAo-injury, *n* = 7) for TOTAL HINDLIMB + FORELIMB movement **(A)**, HINDLIMB + COMPLEX FORELIMB movement **(B)** and HINDLIMB + SIMPLE FORELIMB movement **(C)**. Mean values ± SD and individual points are provided in panels A-C. MCAo-injury reduced the neocortical area for total HINDLIMB-FORELIMB movement and for HINDLIMB + COMPLEX FORELIMB movement; representative images are provided for sham-injury **(D)** and MCAo-injury **(E)**. Correlation testing was performed between final forelimb reaching performance on SPR (average of final 4 sessions) relative to all tested properties of neocortical movement derived from LD-ICMS. Two positive relationships were significantly and positively correlated with final SPR behavior: the area of complex RETRACT **(F)** and TOTAL HINDLIMB-FORELIMB movement **(G)**. *P ≤ 0.05, ^**^P ≤ 0.01. Bregma is represented as a 0 value in the anterior-posterior axis.

**FIGURE 5:**
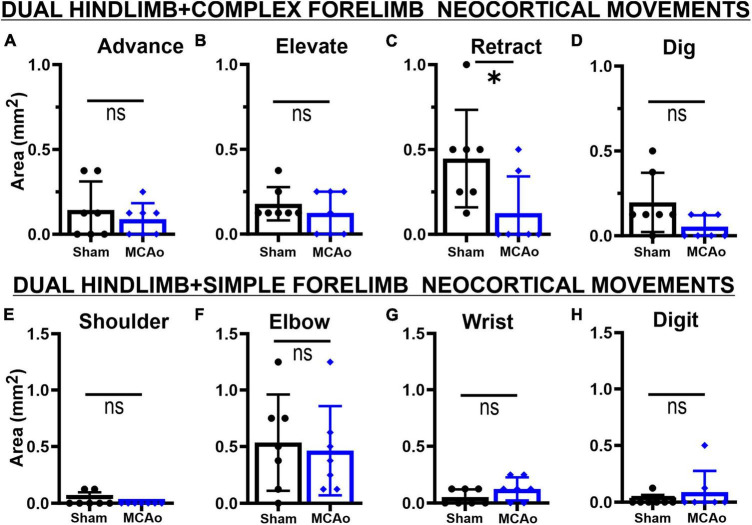
Co-occurring neocortical RETRACT + HINDLIMB movement is uniquely decreased in MCAo animals. LD-ICMS-evoked dual HINDLIMB + FORELIMB movements were subdivided by type of complex forelimb **(A–D)** or type of simple movement **(E–H)** movement. Bar graphs represent mean values ± SD of each movement type (that co-occurs with HINDLIMB), in each experimental group, and individual data points. The co-occurrence of complex movements of ADVANCE **(A)**, ELEVATE **(B)**, and DIG **(D)** with HINDLIMB exhibited no significant difference between sham-injured (*n* = 7) and MCAo-injured groups (*n* = 7). In contrast, co-occurrence of complex RETRACT with HINDLIMB **(C)** demonstrated a significant reduction in area in the MCAo-injured group. No significant effects were observed for the co-occurrence of simple movements of SHOULDER **(E)**, ELBOW **(F)**, WRIST **(G)** and DIGIT **(H)** with HINDLIMB between sham-injured (*n* = 7) and MCAo-injured groups (*n* = 7). *P ≤ 0.05.

As it has previously been reported that interactions between forelimb and hindlimb neocortical networks may contribute to recovery from ischemic injury (Starkey et al., 2012), we quantified all hindlimb movement responses that co-occurred at sites presenting forelimb movement (simple or complex). Neocortical area of FORELIMB + HINDLIMB overlap quantified was for total forelimb, total complex forelimb, total simple forelimb (Figures 4A–E) in addition to their respective movement sub-types (Figure 5). The area of overlapping FORELIMB + HINDLIMB neocortex, for total forelimb output, was significantly reduced [*T*(12) = 1.81; *p* = 0.048] in the MCAo group (mean: 1.13 ± 0.26) compared to the Sham-Injured group (mean: 0.089 ± 0.053) (Figures 4A, D, E). The area of overlapping FORELIMB + HINDLIMB neocortex, for complex forelimb output, was also significantly reduced [*T*(12) = 3.73; *p* = 0.002] in the MCAo group (mean: 0.45 ± 0.13) compared to the Sham-Injured group (mean: 1.05 ± 0.11) (Figure 4B).

In contrast, area of simple forelimb output as overlapping FORELIMB + HINDLIMB neocortex was not significantly different between the MCAo-Injured group (mean: 0.68 ± 0.22) in comparison to Sham-Injured controls (mean: 0.64 ± 0.20) [*T*(12) = 0.12; *p* = 0.45] (Figure 4C). Correlational testing was performed to identify any complex or simple neocortical properties that paralleled final behavioral recovery (as determined by the SPR task) (Figures 4F, G). Two significant positive correlations were detected between neocortical motor output and final SPR behavior: the area of complex RETRACT (*R*^2^ = 0.44, *p* = 0.011; Figure 4F) and area of TOTAL HINDLIMB + FORELIMB movement (*R*^2^ = 0.36, *p* = 0.031; Figure 4G).

Regarding area of overlapping FORELIMB + HINDLIMB regions of neocortex, the individual complex and simple neocortical movements were also investigated (Figure 5). For area of respective movement sub-types for complex forelimb movement, RETRACT was the only individual type of FORELIMB + HINDLIMB overlap that was reduced in the MCAo group (mean: 0.13 ± 0.082) compared to the Sham-Injured group (mean: 0.45 ± 0.109) [*T*(12) = 2.36; *p* = 0.018] (Figure 5C). No significant effects were detected for FORELIMB + HINDLIMB overlap for area of ADVANCE [*T*(12) = 0.73; *p* = 0.24], ELEVATE [*T*(12) = 0.89; *p* = 0.20], and DIG [*T*(12) = 2.021; *p* = 0.033, Mann-Whitney *p* = 0.061] (Figures 5A, B, D). Mean values of the MCAo-Injured group were: ADVANCE: 0.089 ± 0.036; ELEVATE: 0.13 ± 0.047; DIG: 0.054 ± 0.025). Mean values of the Sham-Injured group were: ADVANCE: 0.14 ± 0.064, ELEVATE: 0.18 ± 0.037, DIG: 0.20 ± 0.066.

Regarding area of respective movement sub-types for simple forelimb movement, none of the simple FORELIMB + HINDLIMB overlap areas were significantly reduced with the MCAo-Injury: SHOULDER [*T*(12) = 1.55; *p* = 0.074, Mann-Whitney *p* = 0.23], ELBOW [*T*(12) = 0.33; *p* = 0.38], WRIST [*T*(12) = 1.55; Mann-Whitney *p* = 0.074], and DIGIT [*T*(12) = 0.98; *p* = 0.17, Mann-Whitney *p* = 0.37] (Figures 5E–H). Mean values of the MCAo-Injured group were: SHOULDER: 0.00 ± 0.00 (no sites detected); ELBOW: 0.46 ± 0.15; WRIST: 0.13 ± 0.038; DIGIT: 0.089 ± 0.071). Mean values of the Sham-Injured group were SHOULDER: 0.036 ± 0.023; ELBOW: 0.54 ± 0.16, WRIST: 0.054 ± 0.025; DIGIT: 0.018 ± 0.018.

## Discussion

The present study examined types of complex movement, and their neocortical topography, during forelimb motor recovery from permanent ischemic injury using MCAo. In this study, the neocortex consistently produced 4 types of complex movement involving multiple joints (ADVANCE, ELEVATE, RETRACT AND DIG) and 4 types of simple movement involving single joints (DIGIT, WRIST, ELBOW, SHOULDER) whereas additional test sites were non-forelimb or non-responsive. Test sites co-expressing FORELIMB + HINDLIMB were also fully quantified (Brown et al., 2023). Here, MCAo-Injury induced persistent behavioral deficits in forelimb skilled reaching. MCAo-Injury also differentially affected the neocortical topography of these distinct complex movements at 6 weeks post-injury; wherein, a few of the observed changes exhibited positive correlations with final forelimb behavioral performance. In brief, there was a significant reduction in total neocortical area for complex movement that included reductions in area of ADVANCE, RETRACT and DIG whereas ELEVATE remained statistically unaffected. There was a parallel increase in total neocortical area for simple movement; however, neocortical area for individual simple movement types was numerically but not statistically increased after MCAo. Significant positive correlations were detected between final SPR behavioral performance and either area of complex RETRACT or area of co-occurring complex FORELIMB + HINDLIMB sites.

In this study the reduction in neocortical area of complex motor outputs at 6 weeks post-MCAo was paralleled by an increase in neocortical area of simple motor outputs thereby suggesting an intriguing collapse of this structure’s ability to produce coordinated movement that may underlie certain motor deficits and their treatment-resistant effects after stroke. Behavioral recovery after stroke may be due to the reinstatement of movement and/or the use of compensatory movements. Further testing is needed to identify the mechanistic underpinnings of these changes and to identify targets to therapeutically prevent or reverse these effects of MCAo-Injury. These knowledge gaps will need to be addressed with advanced technologies regarding the anatomy and physiology of the neocortex and corticospinal system that include sophisticated microscopy techniques and neural circuit mapping. These efforts may be bolstered by genetic and viral circuit tracing as well as light sheet microscopy to visualize these relatively long-distance signaling modalities. There also remains a need to further investigate quantitative measures of movement, e.g., video-tracking kinematics, in addition to muscle recruitment, e.g., electromyography.

In the present study, final forelimb behavioral performance correlated with 2 separate types of complex motor output: area of RETRACT and area of combined FORELIMB + HINDLIMB movements. These findings are the first, to the best of our knowledge, to identify these behaviorally relevant changes of complex neocortical motor output following experimental MCAo in mice. RETRACT may correlate with task performance because it represents a key motor component of the SPR reaching movement. Consistent with these findings, (Ramanathan et al., 2006) previously reported in rats that area of RETRACT positively correlated with forelimb motor recovery following a bilateral electrocauterization injury to this region (Ramanathan et al., 2006). Similarity between this published study and the present findings suggests that the brain’s ability to produce specific types of coordinated movement may be critical for motor recovery after brain insult. Furthermore, the neocortical territory for complex RETRACT shares a common topographical border with zones of HINDLIMB, as well as zones of FORELIMB + HINDLIMB overlap, and is in close spatial proximity to parietal cortex in ways that may support behavioral recovery (Neafsey, 1990; Tennant et al., 2011; Brown et al., 2023). RETRACT’s spatial location and properties may underpin its ability to provide anatomical rearrangement of specific neural circuits implicated in stroke recovery however other neural regions are likely to be critical in these processes.

In line with the aforementioned discussion, it remains important to determine whether the capacity for vicariation of motor output (lost by ischemic injury or disease) is due to changes within topographical regions of forelimb, particularly RETRACT, or due to changes in the region of neocortex that borders FORELIMB + HINDLIMB control and additional brain structures. To this point, Starkey et al. (2012) have reported that hindlimb corticospinal neurons appear capable of producing forelimb function following ischemic injury (in rats) based on ischemic damage modeled by intracortical endothelin-1 injections into neocortex. These authors observed a positive correlation between behavioral recovery and the neocortical region occupied by FORELIMB + HINDLIMB overlap that they suggest may be due to direct axon collaterals of hindlimb corticospinal neurons into the cervical spinal cord (Starkey et al., 2012). The capacity of individual corticospinal cells to signal to multiple downstream motor areas, including crucial spinal motor areas, remains an important research topic to understand behavioral recovery in many motor-related diseases and injuries. The similar observations here, relative to Ramanathan et al. (2006) and Starkey et al. (2012), may speak to the robustness of these key features of complex neocortical movement for recovery from brain injury and this study further indicates that shifts in the proportion of complex and simple neocortical motor output may be critical in these processes.

The relationship between complex and simple neocortical motor output remains understudied and may offer promising new approaches for the treatment of long-term brain repair. Simple movements may be components of individual complex movements, or alternatively, may exist as unique motor outputs that are organizationally inborn to the organism. From a developmental perspective the post-natal day (PND) expression of LD-ICMS simple movements (PND 25) precedes that of complex movements (PND30); this relationship is maintained during acute cortical disinhibition using blockade of type A GABA receptors with bicuculline: LD-ICMS simple movements (PND15) precede complex movements (PND20) (Singleton et al., 2021). Calcium imaging in zebrafish finds that developmental motor activity patterns extend from individual motor neurons, to the recruitment of neighboring cells, in a manner that appears to help create neural networks and eventually reach the level of bilaterality (Wan et al., 2019). Indeed, there is a transition between PND8-to-PND12 in rats wherein the neocortex appears to rely upon early simple motor outputs to shift to a more distributed complex network that encodes kinematic features for mature self-guided movements (Glanz et al., 2021).

Complex movements in adult rats do demonstrate reorganization following injury and rehabilitative training in a task-dependent manner to promote functional recovery (Ramanathan et al., 2006; Starkey et al., 2012). Uninjured adult rats respond to SPR training with a significant increase in forelimb sites that co-express non-forelimb movement (Brown and Teskey, 2014). Budri et al. (2014), testing sensorimotor deprivation due to forelimb cast immobility, identified a resulting reduction of neocortical area for complex motor output that was surprising reversible following chronic cast removal. Thus, while the cortical topography of complex forelimb movements may be more stable in adulthood, perhaps due to more established corticospinal topography, physiology and projection patterns (Harrison et al., 2012; Wang et al., 2017), complex movement representations in the adult do exhibit plasticity after injury and experience (Ramanathan et al., 2006; Starkey et al., 2012; Brown and Teskey, 2014; Budri et al., 2014). Here, we extend the finding of complex forelimb movement representation plasticity to the adult mouse after MCAo-Injury and SPR.

Whether complete reversibility of complex motor function is possible after injury remains debatable. The shared observations between the present study and the cited literature, regarding main effects of neocortical complex movement following insult and its correlational relationships with behavioral performance, support the potential capacity for this biology to help aid restoration of motor function. Further testing is needed to determine the uniformity of this restoration, its temporal and spatial dynamics, specificity to motor rehabilitation, responsiveness to separate insults, and cell-type involvement, among other key properties. There remains an urgent need to understand how spared brain regions contribute to functional motor recovery after stroke given that cellular and behavioral recovery often remain incomplete in clinical populations (Tsao et al., 2022). The process of aging is an important consideration since the incidence of stroke increases with age and aging may limit beneficial neuroplastic change in spared brain regions after injury. These treatment-focused research questions will require sophisticated *in vivo*, *in vitro* and *in silico* approaches in concert with long-standing neurophysiological techniques to rapidly provide pre-clinical advancements and optimization of human care.

## Data availability statement

The original contributions presented in this study are included in the article/supplementary material, further inquiries can be directed to the corresponding author.

## Ethics statement

This animal study was reviewed and approved by the University of Texas Health Science Center at San Antonio (UTHSCSA) Institutional Animal Care and Use Committee.

## Author contributions

CW, RB, AB, and JB conceived, designed, and conducted the research. GP contributed to the data analysis and figures preparation. CW, RB, AB, and JB analyzed the data and prepared the figures wherein this effort was spearheaded by CW. CW, RB, and JB drafted the manuscript. All authors revised and approved final version of the manuscript.

## References

[B1] Abràmoff M. D. Magalhães P. J. Ram S. J. (2004). Image processing with ImageJ. *Biophoton. Int.* 11 36–42.

[B2] Aflalo T. N. Graziano M. S. A. (2006). Possible origins of the complex topographic organization of motor cortex: Reduction of a multidimensional space onto a two-dimensional array. *J. Neurosci.* 26:6288. 10.1523/JNEUROSCI.0768-06.2006 16763036 PMC6675193

[B3] Asanuma H. Stoney S. D. Jr. Abzug C. (1968). Relationship between afferent input and motor outflow in cat motorsensory cortex. *J. Neurophysiol.* 31 670–681. 10.1152/jn.1968.31.5.670 5711138

[B4] Baldwin M. K. L. Cooke D. F. Goldring A. B. Krubitzer L. (2018). Representations of fine digit movements in posterior and anterior parietal cortex revealed using long-train intracortical microstimulation in macaque monkeys. *Cereb. Cortex* 28 4244–4263. 10.1093/cercor/bhx279 29136133 PMC6215470

[B5] Baldwin M. K. Cooke D. F. Krubitzer L. (2017). Intracortical microstimulation maps of motor, somatosensory, and posterior parietal cortex in tree shrews (*Tupaia belangeri*) reveal complex movement representations. *Cereb. Cortex* 27 1439–1456. 10.1093/cercor/bhv329 26759478 PMC6075024

[B6] Bonazzi L. Viaro R. Lodi E. Canto R. Bonifazzi C. Franchi G. (2013). Complex movement topography and extrinsic space representation in the rat forelimb motor cortex as defined by long-duration intracortical microstimulation. *J. Neurosci.* 33 2097–2107. 10.1523/JNEUROSCI.3454-12.2013 23365246 PMC6619127

[B7] Boychuk J. A. Adkins D. L. Kleim J. A. (2011). Distributed versus focal cortical stimulation to enhance motor function and motor map plasticity in a rodent model of ischemia. *Neurorehabil. Neural Repair* 25 88–97. 10.1177/1545968310385126 21062949

[B8] Boychuk J. A. Farrell J. S. Palmer L. A. Singleton A. C. Pittman Q. J. Teskey G. C. (2017). HCN channels segregate stimulation-evoked movement responses in neocortex and allow for coordinated forelimb movements in rodents. *J. Physiol.* 595 247–263. 10.1113/JP273068 27568501 PMC5199725

[B9] Brown A. R. Teskey G. C. (2014). Motor cortex is functionally organized as a set of spatially distinct representations for complex movements. *J. Neurosci.* 34 13574–13585. 10.1523/JNEUROSCI.2500-14.2014 25297087 PMC6608383

[B10] Brown A. R. Coughlin G. M. Teskey G. C. (2020). Seizures alter cortical representations for complex movements. *Neuroscience* 449 134–146. 10.1016/j.neuroscience.2020.09.002 32916196

[B11] Brown A. R. Mitra S. Teskey G. C. Boychuk J. A. (2023). Complex forelimb movements and cortical topography evoked by intracortical microstimulation in male and female mice. *Cereb. Cortex* 33 1866–1875. 10.1093/cercor/bhac178 35511684 PMC9977357

[B12] Budri M. Lodi E. Franchi G. (2014). Sensorimotor restriction affects complex movement topography and reachable space in the rat motor cortex. *Front. Syst. Neurosci.* 8:231. 10.3389/fnsys.2014.00231 25565987 PMC4264501

[B13] Cooke D. F. Padberg J. Zahner T. Krubitzer L. (2012). The functional organization and cortical connections of motor cortex in squirrels. *Cereb. Cortex* 22 1959–1978. 10.1093/cercor/bhr228 22021916 PMC3412438

[B14] Cooke D. F. Stepniewska I. Miller D. J. Kaas J. H. Krubitzer L. (2015). Reversible deactivation of motor cortex reveals functional connectivity with posterior parietal cortex in the prosimian galago (*Otolemur garnettii*). *J. Neurosci.* 35 14406–14422. 10.1523/JNEUROSCI.1468-15.2015 26490876 PMC4683694

[B15] Gharbawie O. A. Stepniewska I. Kaas J. H. (2011a). Cortical connections of functional zones in posterior parietal cortex and frontal cortex motor regions in new world monkeys. *Cereb. Cortex* 21 1981–2002. 10.1093/cercor/bhq260 21263034 PMC3155600

[B16] Gharbawie O. A. Stepniewska I. Qi H. Kaas J. H. (2011b). Multiple parietal-frontal pathways mediate grasping in macaque monkeys. *J. Neurosci.* 31 11660–11677. 10.1523/JNEUROSCI.1777-11.2011 21832196 PMC3166522

[B17] Glanz R. M. Dooley J. C. Sokoloff G. Blumberg M. S. (2021). Sensory coding of limb kinematics in motor cortex across a key developmental transition. *J. Neurosci.* 41 6905–6918. 10.1523/JNEUROSCI.0921-21.2021 34281990 PMC8360693

[B18] Glees P. Cole J. (1949). The reappearance of coordinated movements of the hand after lesions in the hand area of the motor cortex of the rhesus monkey. *J. Physiol.* 108:33. 18200702

[B19] Graziano M. S. (2016). Ethological action maps: A paradigm shift for the motor cortex. *Trends Cogn. Sci.* 20 121–132. 10.1016/j.tics.2015.10.008 26628112

[B20] Graziano M. S. Aflalo T. N. Cooke D. F. (2005). Arm movements evoked by electrical stimulation in the motor cortex of monkeys. *J. Neurophysiol.* 94 4209–4223. 10.1152/jn.01303.2004 16120657

[B21] Graziano M. S. Taylor C. S. Moore T. (2002). Complex movements evoked by microstimulation of precentral cortex. *Neuron* 34 841–851. 10.1016/S0896-6273(02)00698-0 12062029

[B22] Griffin D. M. Hudson H. M. Belhaj-Saïf A. Cheney P. D. (2014). EMG activation patterns associated with high frequency, long-duration intracortical microstimulation of primary motor cortex. *J. Neurosci.* 34 1647–1656. 10.1523/JNEUROSCI.3643-13.2014 24478348 PMC3905140

[B23] Halley A. C. Baldwin M. K. L. Cooke D. F. Englund M. Krubitzer L. (2020). Distributed motor control of limb movements in rat motor and somatosensory cortex: The sensorimotor amalgam revisited. *Cereb. Cortex* 30 6296–6312. 10.1093/cercor/bhaa186 32691053 PMC8248848

[B24] Harrison T. C. Ayling O. G. Murphy T. H. (2012). Distinct cortical circuit mechanisms for complex forelimb movement and motor map topography. *Neuron* 74 397–409. 10.1016/j.neuron.2012.02.028 22542191

[B25] Kleim J. A. Barbay S. Nudo R. J. (1998). Functional reorganization of the rat motor cortex following motor skill learning. *J. Neurophysiol.* 80 3321–3325. 10.1152/jn.1998.80.6.3321 9862925

[B26] Liepert J. Graef S. Uhde I. Leidner O. Weiller C. (2000). Training-induced changes of motor cortex representations in stroke patients. *Acta Neurol. Scand.* 101 321–326. 10.1034/j.1600-0404.2000.90337a.x 10987321

[B27] Mayer A. Baldwin M. K. L. Cooke D. F. Lima B. R. Padberg J. Lewenfus G. (2019). The multiple representations of complex digit movements in primary motor cortex form the building blocks for complex grip types in capuchin monkeys. *J. Neurosci.* 39 6684–6695. 10.1523/JNEUROSCI.0556-19.2019 31235643 PMC6703879

[B28] Neafsey E. J. Bold E. L. Haas G. Hurley-Gius K. M. Quirk G. Sievert C. F. (1986). The organization of the rat motor cortex: A microstimulation mapping study. *Brain Res.* 396 77–96. 10.1016/0165-0173(86)90011-1 3708387

[B29] Neafsey J. (1990). “The complete ratunculus: Output organization of layer V of the cerebral cortex,” in *The cerebral cortex of the rat*, eds Kolb B. Tees R. C. (Cambridge, MA: MIT), 197–212.

[B30] Nudo R. J. Wise B. M. Sifuentes F. Milliken G. W. (1996). Neural substrates for the effects of rehabilitative training on motor recovery after ischemic infarct. *Science* 272 1791–1794. 10.1126/science.272.5269.1791 8650578

[B31] Ramanathan D. Conner J. M. Tuszynski M. H. (2006). A form of motor cortical plasticity that correlates with recovery of function after brain injury. *Proc. Natl. Acad. Sci. U.S.A.* 103 11370–11375. 10.1073/pnas.0601065103 16837575 PMC1544093

[B32] Singleton A. C. Brown A. R. Teskey G. C. (2021). Development and plasticity of complex movement representations. *J. Neurophysiol.* 125 628–637. 10.1152/jn.00531.2020 33471611

[B33] Starkey M. L. Bleul C. Zörner B. Lindau N. T. Mueggler T. Rudin M. (2012). Back seat driving: Hindlimb corticospinal neurons assume forelimb control following ischaemic stroke. *Brain* 135 3265–3281. 10.1093/brain/aws270 23169918

[B34] Stepniewska I. Fang P.-C. Kaas J. H. (2005). Microstimulation reveals specialized subregions for different complex movements in posterior parietal cortex of prosimian galagos. *Proc. Natl. Acad. Sci. U.S.A.* 102 4878–4883. 10.1073/pnas.0501048102 15772167 PMC555725

[B35] Stepniewska I. Gharbawie O. A. Burish M. J. Kaas J. H. (2014). Effects of muscimol inactivations of functional domains in motor, premotor, and posterior parietal cortex on complex movements evoked by electrical stimulation. *J. Neurophysiol.* 111 1100–1119. 10.1152/jn.00491.2013 24353298 PMC3949230

[B36] Tennant K. A. Adkins D. L. Donlan N. A. Asay A. L. Thomas N. Kleim J. A. (2011). The organization of the forelimb representation of the C57BL/6 mouse motor cortex as defined by intracortical microstimulation and cytoarchitecture. *Cereb. Cortex* 21 865–876. 10.1093/cercor/bhq159 20739477 PMC3059888

[B37] Tsao C. W. Aday A. W. Almarzooq Z. I. Alonso A. Beaton A. Z. Bittencourt M. S. (2022). Heart disease and stroke statistics—2022 update: A report from the American Heart Association. *Circulation* 145 e153–e639. 10.1161/CIR.0000000000001052 35078371

[B38] Wan Y. Wei Z. Looger L. L. Koyama M. Druckmann S. Keller P. J. (2019). Single-cell reconstruction of emerging population activity in an entire developing circuit. *Cell* 179 355–372.e323. 10.1016/j.cell.2019.08.039 31564455 PMC7055533

[B39] Wang X. Liu Y. Li X. Zhang Z. Yang H. Zhang Y. (2017). Deconstruction of corticospinal circuits for goal-directed motor skills. *Cell* 171 440–455.e414. 10.1016/j.cell.2017.08.014 28942925 PMC5679421

[B40] Whishaw I. Q. Pellis S. M. (1990). The structure of skilled forelimb reaching in the rat: A proximally driven movement with a single distal rotatory component. *Behav. Brain Res.* 41 49–59. 10.1016/0166-4328(90)90053-H 2073355

[B41] Young N. A. Vuong J. Flynn C. Teskey G. C. (2011). Optimal parameters for microstimulation derived forelimb movement thresholds and motor maps in rats and mice. *J. Neurosci. Methods* 196, 60–69. 10.1016/j.jneumeth.2010.12.028 21219927

